# Challenges to access health information during pregnancy in Iran: a qualitative study from the perspective of pregnant women, midwives and obstetricians

**DOI:** 10.1186/s12978-019-0789-3

**Published:** 2019-08-22

**Authors:** Marzieh Javanmardi, Mahnaz Noroozi, Firouzeh Mostafavi, Hasan Ashrafi-rizi

**Affiliations:** 1 School of Nursing and Midwifery, Isfahan University of Medical Sciences, Isfahan, Iran; 2 Department of Midwifery and Reproductive Health, School of Nursing and Midwifery, Isfahan University of Medical Sciences, Isfahan, Iran; 3 Department of Health Education and Promotion, School of Health, Isfahan University of Medical Sciences, Isfahan, Iran; 4 Library and Information Science, Health Information Technology Research Center, Isfahan University of Medical Sciences, Isfahan, Iran

**Keywords:** Information seeking behavior, Pregnant women, Barriers, Qualitative study

## Abstract

**Background:**

Appropriate health information seeking behavior can play an effective role in self-care and promotion of women’s quality of life during pregnancy. However, different barriers can impede pregnant women while accessing health information. The aim of this research was to explain challenges to access health information during pregnancy.

**Methods:**

The present qualitative study was carried out on 28 participants who were selected using the purposeful sampling technique. Data were collected through in-depth interviews, field notes, and daily notes; data were analyzed using conventional content analysis.

**Results:**

The main barriers to access health information during pregnancy were as follows: many duties of women at home as well as out-of-home education and employment, inability to make distinction between correct and incorrect information, insufficient interactions between women and healthcare providers, failure to access to various information resources, common complaints of pregnancy, and stress and anxiety of confronting the problems during pregnancy.

**Conclusion:**

Based on the results, pregnant women experienced personal, social, and structural barriers when accessing health information. Therefore, policymakers and health planners should remove the barriers, encourage self-care, and enhance the quality of life for pregnant women, thus, promoting their health status in the end.

## Plain English summary

Pregnant women need health information to increase their empowerment while practicing preventive health behaviors and boosting self-care capabilities. Although women desire to receive information during pregnancy, they are often faced with challenges when accessing health information; therefore, this study was conducted to explain challenges to access health information during pregnancy. Results in this field were achieved through interviews with pregnant women and healthcare providers (midwives and obstetricians). The top six research themes identified were: many duties of women at home as well as out-of-home education and employment, inability to make distinction between correct and incorrect information, insufficient interactions between women and healthcare providers, failure to access to various information resources, common complaints of pregnancy, and stress and anxiety of confronting the problems during pregnancy. In conclusion, these results could be helpful for health policymakers in designing strategies to increase the access of pregnant women to health information, thereby, promoting their self-care capability, and quality of life, which will ultimately improve the health of the mothers.

## Background

Information seeking is a complex activity that requires access to various information resources to solve personal, social, and occupational problems. A review of related literatures on information needs and health information seeking behavior (HISB) revealed more attention to a specific user groups in recent years [1]. One of these groups that are of great importance in terms of their information seeking includes women. Undoubtedly, women’s health and their updated level of awareness and knowledge will have tremendous effect on society [2]. Pregnancy is not only a period of physical changes; it is also a phase in the life of a woman where health-related behaviors are critical to decisions made and can affect the life of the mother and her neonatal outcomes [3]. Pregnant women need health information to increase their empowerment while practicing preventive health behaviors, boosting self-care capabilities, and reducing anxiety in case of new health issues or stressful situations [4]. Merrell et al. showed that exploring HISB of pregnant women is the first step in understanding the health literacy in this significant population [5]. In this regard, researchers believed that healthcare providers should allocate efficient amount of time for discussing information-seeking methods with pregnant women [6].

The results of some studies suggested that although women desire to receive information during pregnancy, they are often faced with challenges when accessing health information [1, 2]. Nwagwu and Ajama pointed out to factors such as distance, high costs, and language differences as the barriers that pregnant women face when accessing health information [7]^.^ Das and Sarka stated in their study that pregnant women experienced different barriers when accessing health information; these challenges include poor quality of care provided in the hospital, long waiting time, fear and embarrassment to discussing pregnancy with a physician and shortage of time [8]. Although providing pregnant women with health information is a critical component of the process of pregnancy management, there is no leading national health policy and strategy to promote health information system for women during pregnancy in Iran. Understanding the nature of obstacles for health information seeking will help healthcare policymakers to provide evidence-based and reliable information to encourage pregnant women involvement in management and treatment decisions. Although the importance of this issue cannot be overemphasized, studies on HISB in pregnant women are a few in Iran [1, 9, 10]. Since qualitative research is an approach to discover and describe the experiences of individuals and give meaning to them, on the one hand, it leads to increased insight, understanding, and awareness of human experiences. On the other hand, it is used to explain the concepts and relationships between them [11]. Hence, the aim of this research was to explain challenges to access health information during pregnancy.

## Methods

### Study design

This study is a qualitative research that was conducted from June to November 2016*.*

### Settings, sample, and recruitment

The approval letter of the research was obtained from the Ethics Committee of the Vice-Chancellor of Research of Isfahan University of Medical Sciences (Ethics code: IR.MUI.REC.1395.3.955). Participants in the present study were 22 pregnant women and six healthcare providers (midwives and obstetricians) in Isfahan, Iran; which were selected through purposive sampling method. The pregnant women were selected with a maximum variation sampling strategy based on age, level of education, job status, gestational age, and number of pregnancies and the ability to understand and express their experiences. Participants were accessed through prenatal clinics, healthcare centers, and midwife or obstetrician offices. They were recruited through face-to-face meeting or telephone calls. The first author (MJ) did not have any role or relationship with the centers or participants.

### Data collection

Semi-structured in- depth interviews, field notes and daily notes were used to collect data. The time and place of the interviews were determined based on the convenience of the participants. Considering the participants were required to have first-hand experiences and information about the research topic in the qualitative research [11], firstly, interviews carried out on pregnant women. In interview sessions, questions were different for the each group of the participants. Interviews with pregnant women were started by posing a general question “Please explain the barriers you faced while seeking the health information during pregnancy?” and then the open and interpretive responses of the participants guided its process. Interviews with other participants (midwives and obstetricians) were begun by posing another general question “Based on your experiences, please explain the barriers that pregnant women faced while seeking the health information during pregnancy?” Since the official language in Iran is Persian, the specific language used for data collection was Persian. The interviews lasted between 30 and 90 min and were recorded using a digital voice recorder. Interviews continued until data saturation point was reached, i.e. when new findings were not added to the existing data.

In this study, the first author (MJ) documented her observations of the non-verbal behaviors of participants during the interview (field notes). Participants were also asked to note, if they were willing, their information requirements, sources of information, and problems in acquiring health information during pregnancy and to hand these notes (daily notes) to the first author (MJ). In the present study, obtaining informed consent, the right of anonymity, confidentiality of the information and the right of withdrawal from the study at any desired time were respected.

### Data analysis

The data were analyzed manually and the authors did not use any type of software for analysis. Data analysis was carried out using the conventional content analysis method [12]. Interviews were transcribed verbatim and were repeatedly reviewed to achieve a complete understanding. Then, the sentences and phrases were coded and the same codes were merged and the ones having the same concept were placed in the same category (in the inductive manner), which formed the sub-categories and eventually the main category.

### Rigor and trustworthiness

To ensure the credibility of the findings, various methods including in-depth interviews at different times and places, a combination of several data collection methods such as individual interviews, daily notes, and field notes were applied. To ensure the confirmability and to approve the accuracy of the codes and interpretations, it was shared with five participants at different sessions and their opinions were sought to conduct a member check. To ensure dependability, the views of four experts were also used to match the consistency of the findings with the statements of the participants. To ensure the transferability, the findings of the present study were presented to 11 individuals who did not participate in the study, but had characteristics similar to the participants to judge the similarity of the results of the study with their own experiences.

## Results

The demographic characteristics of the 28 participants are shown in Table 1. During data analysis, 84 codes, six sub-categories, and one main category developed. Six sub-categories were as follows: “insufficient interactions between pregnant women and healthcare providers”, “stress and anxiety of confronting the problems”, “pregnancy-related common complaints”, “busy life and shortage of time”, “problems with access to various information resources” and “failure to make distinction between correct and incorrect information” (Table 2).

**Table 1:** Demographic characteristics of participants

Pregnant women (*n* = 22)	Age (years)	16–35
	Level of education	Secondary (4), Diploma (5), Associate’s degree and B.S. (11), M.S. and Ph.D. (2)
	Job status	Employed (7), Housewife (15)
	Pregnancy trimester	First trimester (3), Second trimester (8), Third trimester (11)
	Pregnancy number	1–3
Healthcare providers (*n* = 6)	Age (years)	35–47
	Working experience (years)	5–24

**Table 2:** The examples of codes, sub-categories and main category

Code	Sub-category	Main category
* Failure to receive adequate information through an obstetrician and midwife * Failure to consult to pregnant women via phone call * The crowds of obstetrician office and the lack of enough time to visit clients	Insufficient interactions between pregnant women and healthcare providers	The challenges to access health information during pregnancy
* Fear of complication during pregnancy * Creating fear after observing normal vaginal delivery movies * Making worries due to received information from family members and relatives * Fear following by receiving information on how to get normal delivery	Stress and anxiety of confronting the problems	
* Nausea and vomiting in pregnancy * Feeling boredom in pregnancy * Feeling tired in pregnancy	Pregnancy-related common complaints	
* Out-of-home employment and lack of time * Education in college and not having enough time * Taking care of the children * Busy life and several duties and responsibilities in home	Busy life and shortage of time	
* The high cost of buying books * The lack of up-to-date libraries * Trouble in finding the book	Problems with access to various information resources	
* Non-scientific information received from family members and relatives * Confusion after observation of conflicting information on websites	Failure to make distinction between correct and incorrect information	

### Insufficient interactions between pregnant women and healthcare providers

As noted by the participating pregnant women, they trusted the information provided by obstetricians and midwives and considered them as a reliable source for their information needs. However, most of them were dissatisfied with the short-term visits by the obstetricians (due to workload), the presence of several pregnant women in the visiting room at the same time and providing the insufficient information. They believed the afore-mentioned items were barriers to accessing health information. In addition, a number of pregnant women considered the young age of healthcare providers or the fact that they are un-married as barriers in communicating appropriately with them to receive information about sexual matters during pregnancy.

*“I think they (obstetricians) could not manage us very well and they do not talk with us much about our pregnancy” (Pregnant woman, 29 years old).*

*“The level of knowledge of pregnant women is very low. The obstetricians only give them simple prenatal care. … Pregnant women have lots of questions when they go to a health center, but we do not have enough time to answer them properly” (Midwife, 35 years old).*

### Stress and anxiety of confronting the problems

Some pregnant women said that consultation with family members and relatives made them anxious and thus they tend to consult with an obstetrician or midwife for information instead. Some pregnant women stated that obtaining information about warning signs in pregnancy, complications of pregnancy, and normal delivery (through watching movies) were factors causing stress and fear of confronting these problems. They believed that familiarity with these issues increased their sensitivity and it would make them anxious with the smallest symptom. They considered this stress as a barrier to seek more information.

*“When I got informed, it would make me distressed. Sometimes, I decided to be ignorant and less informed. I told to myself: ‘It’s better not to get informed!’ if makes me frustrated, I would rather not know them at all …*” *(Pregnant woman, 31 years old).*

### Pregnancy-related common complaints

Some pregnant women referred to common complaints of pregnancy as the barriers to accessing health information. Among the complaints frequently noted by the majority of participants included: nausea and vomiting, which, as stated by them, disrupted their daily routine activities and made them became more bored. Some pregnant women also stated that fatigue, and the presence of sleep problems, followed by lethargy throughout the day would prevent them from meeting their information needs.

*“I was sometimes tired of everything due to difficulties of pregnancy (morning sickness) and I wished I had not been pregnant at all and never followed it accordingly (seeking information)” (Pregnant woman, 23 years old).*

### Busy life and shortage of time

Some pregnant women referred to the duties of women at home and taking care of the spouse and other children as a barrier to seeking information, while others stated that it was not possible for them to participate in “childbirth preparation classes” and refer to healthcare centers because of their out-of-home employment. A number of pregnant women also narrated that they did not have enough time to seek health information during pregnancy due to their education.

*“... A woman has many responsibilities: cooking, taking care of the child, taking care of husband, she shoulders all of these (responsibilities). So, there is no time left for her to read books.” (Pregnant woman, 35 years old).*

### Problems with access to various information resources

Pregnant women and healthcare providers referred to difficulty in accessing the various resources, such as insufficient printed materials (book etc.), high cost of printed materials, the lack of up-to-date libraries, and the lack of prenatal educational movies tailored to the culture of society (about exercises in pregnancy etc.), as other barriers for accessing health information during pregnancy. Some pregnant women complained about the lack of possibility to use remote (telephone) counseling.

*“In case of exercises during pregnancy, I searched a lot on the internet. There were some online films, none of which was Iranian... All the existing movies were made by the non-Iranian producers. I was worried if it is ok; because some of them (exercises) were very heavy ...” (Pregnant women, 31 years old).*

### Failure to make distinction between correct and incorrect information

Despite the high and easy use of the internet and social networks, many of pregnant women and healthcare providers pointed to the existence of inaccurate and non-scientific information on various websites and stated that pregnant women were unable to make distinction between correct and incorrect information were received from different sources. Pregnant women sometimes had to use several sources to investigate the validity of the information, or to determine the accuracy of the received information by referring to the health centers and asking the obstetrician or midwife. They also expressed dissatisfaction with the failure to provide reliable sources on behalf of media and healthcare providers.

*“Unfortunately, superstitions, which are commonplace among the common people, have priority and higher effectiveness over the information given by the midwife and the obstetrician, and in my opinion, they believe in the words of others, while the information given by family, mother or sister is non-scientific!!!” (Midwife, 42 years old).*

Many pregnant women expressed concern about wrapping of the umbilical cord around the fetus’s neck and choking, and believed that this is related to the position of the mother during sleep and rest. Healthcare providers believed that misinformation given to pregnant women by different individuals with different experiences, and the inability of women to distinguish between correct and incorrect information can cause anxiety in these women.

*“There are misconceptions about pregnancy among pregnant women that cause their concern. Many mothers believe that squirming in bed would cause the umbilical cord to wrap around the neck of the fetus. Some also believe that raising the hands by the pregnant woman would cause the fetus’s umbilical cord to tear …*” *(Obstetrician, 40 years old).*

## Discussion

The aim of this research was to explain challenges to access health information during pregnancy. Participants in the present study emphasized the insufficient interactions between pregnant women and healthcare providers. Lagan et al. showed that about half of the mothers (48.6%) were dissatisfied with the information provided by healthcare providers, and they made use of the internet because healthcare providers spend insufficient time to provide information [13]. Today, crowded physician’s office and inappropriate methods used for visiting patients in Iran, such as “group visit of patients,” have led obstetricians not to spend enough time to visit pregnant women. This issue has not only led to the reduction of the quality of health services but has also caused an increased level of clients’ dissatisfaction. One of the reasons for “group visit of patients” is the lack of implementation of the referral system for medical services. So, implementation and adherence to the referral system for medical services seems to be the only solution [14–16].

In the present study, pregnant women referred to stress and fear of confronting the problems following information acquisition as another barrier to accessing health information. Feltwell and Rees showed that some individuals were afraid of seeking information and believed that this would increase their concern. They avoided seeking information to reduce their levels of fear and to maintain a sense of normality [17]. Therefore, it is essential to pay attention to stress management using solutions such as psychological supporting and providing education and awareness-raising programs on pregnancy, delivery, and familiarity with the hospital environment, especially labor and delivery room.

In the present study, participants referred to the presence of common complaints such as nausea, vomiting, and fatigue during pregnancy as another barrier to accessing health information. This finding can be justified considering the high prevalence of these problems and their impact on the daily activities of pregnant women [18]. Therefore, measures should be taken to reduce the most common complaints in women during pregnancy so that they can carry out their daily chores and get health information during pregnancy.

The results of the present study showed that women engagement in out-of-home employment as well as doing household chores could limit the opportunity for information seeking. Onuoha and Amuda referred to shortage of time as a challenge to seeking information among pregnant women [2]. It seems that women need special attention and support from the family and the community during pregnancy. This support should be especially given to women who are working and studying.

Participants in the present study referred to problems such as the high cost of printed materials, out-of-date libraries, and lack of prenatal educational movies tailored to community culture as the barriers to accessing health information during pregnancy. Nwagwu and Ajama also pointed to some of these barriers to accessing information such as distance and high cost [7]. Therefore, the health system is needed to find solutions to enhance the welfare of low-income pregnant women and take more serious measures for providing free counseling and health information services for pregnant women through health centers. It is also very useful and effective to provide information services to pregnant women using printed materials. Radio and television, as cheap and easy-accessed resources can be used to transfer information; they can play an important role in removing the above barriers.

In the present study, another barrier to accessing health information was the participants’ unfamiliarity with useful resources and failure to make distinction between correct and incorrect information. According to the participants, plenty of websites caused confusion among pregnant women and; they expressed dissatisfaction with the insignificant role of obstetricians and midwives as well as the media in introducing useful and credible references. Bert et al. reported that some patients wanted to investigate the information received from the internet and the needed to compare this information with other sources [19]. Given the increasing use of internet by pregnant women to obtain health information and the inability of women to assess the validity of internet sources [6, 20, 21], academia, healthcare providers, media, and other actors are required to review the online content, and single out valid websites to introduce them to their clients. In addition, healthcare providers, upon designing valid websites should try to guide women to access high-quality web-based information taking into account the information needs of pregnant women.

The generalization of the results of this qualitative study should be done with caution. Considering qualitative studies do not have any claim regarding generalizability of the findings, it may be important for the people who wish to apply the results of the study and be considered as a limitation. In this regard, efforts were made to increase the rigor and trustworthiness of the findings through the selection of participants with the maximum variations, guidance, and supervision of experts as well as external review.

## Conclusion

The present study revealed insufficient interactions between pregnant women and healthcare providers, stress, and anxiety of confronting the problems, pregnancy-related common complaints, busy life, and shortage of time, problems with access to various information resources and failure to make distinction between correct and incorrect information are as barriers to accessing health information among pregnant women. So, serious efforts should be made to remove these barriers. In this regard, effective solutions should be designed to facilitate the access of pregnant women to health information, thereby, promoting their self-care capability, and quality of life, which will ultimately improve the health of the mothers.

## Data Availability

The datasets generated and analysed during the current research are not publicly available as individual privacy could be compromised but are available from the corresponding author on reasonable request.

## Abbreviation

HISB: Health Information Seeking Behavior
